# Simulation-based clinical systems testing for healthcare spaces: from intake through implementation

**DOI:** 10.1186/s41077-019-0108-7

**Published:** 2019-08-02

**Authors:** Nora Colman, Cara Doughty, Jennifer Arnold, Kimberly Stone, Jennifer Reid, Ashley Dalpiaz, Kiran B. Hebbar

**Affiliations:** 1 Department of Pediatrics, Division of Pediatric Critical Care, Children’s Healthcare of Atlanta, 1405 Clifton Road NE, Atlanta, GA 30329 USA; 2 Section of Emergency Medicine, Department of Pediatrics, Baylor College of Medicine, Texas Children’s Hospital, 6621 Fannin Street, Suite A210, Houston, TX 77030 USA; 3 Department of Pediatrics, Maternal, Fetal, Neonatal Institute, Johns Hopkins All Children’s Hospital, 501 6th Avenue S, St. Petersburg, FL 33701 USA; 4 Department of Pediatrics, Division of Emergency Medicine, Seattle Children’s Hospital and University of Washington School of Medicine, 1959 NE Pacific Street, Seattle, WA 98195 USA; 5 Department of Pediatrics, Children’s Healthcare of Atlanta, 1655 Tullie Circle, Atlanta, GA 30329 USA

**Keywords:** Patient Safety, FMEA, Healthcare design, Latent safety threats

## Abstract

Healthcare systems are urged to build facilities that support safe and efficient delivery of care. Literature demonstrates that the built environment impacts patient safety. Design decisions made early in the planning process may introduce flaws into the system, known as latent safety threats (LSTs). Simulation-based clinical systems testing (SbCST) has successfully been incorporated in the post-construction evaluation process in order to identify LSTs prior to patient exposure and promote preparedness, easing the transition into newly built facilities. As the application of simulation in healthcare extends into the realm of process and systems testing, there is a need for a standardized approach by which to conduct SbCST in order to effectively evaluate newly built healthcare facilities. This paper describes a systemic approach by which to conduct SbCST and provides documentation and evaluation tools in order to develop, implement, and evaluate a newly built environment to identify LSTs and system inefficiencies prior to patient exposure.

**Electronic supplementary material:**

The online version of this article (10.1186/s41077-019-0108-7) contains supplementary material, which is available to authorized users.

## Introduction

Healthcare systems are urged to build facilities that support safe and efficient patient care as well as staff safety [1, 2]. Literature demonstrates that the built environment, defined as the indoor atmosphere, interior design, and relative location of spaces, impacts patient safety [3, 4]. Design decisions made early on can inadvertently introduce system flaws creating latent safety threats (LSTs) and system inefficiencies [4–6]. Given that the volume of new construction and renovation in healthcare are increasing [7], the ability to evaluate and understand the complex interactions of the built environment, people, technology, and equipment must be incorporated into the design process in order to effectively mitigate risks prior to patient exposure [1, 4, 8].

Administrative and operational planning prior to new facility opening asks users of the proposed space to conceptualize and imagine how work should be done [4, 9, 10]. However, work as imagined is often not an accurate reflection of the real conditions that impact patient care [10]. Simulation-based clinical systems testing (SbCST) allows hospital leaders and clinicians to evaluate work as done taking into account human factors and the complex interactions of people with the built environment which makes space utilization and process implementation unpredictable [10].

SbCST in the post-construction phase of design, prior to facility opening and beginning patient care, has been applied to systems to detect latent safety threats [11], ensure operational readiness [12], and ease transitioning healthcare systems by promoting preparedness or training emergency response teams [9, 13–15]. Each study employs a different process for conducting simulation, and without standard documentation and evaluation tools, this makes it difficult to replicate at other institutions and apply to varying healthcare spaces [16]. As the application of simulation in healthcare extends beyond education, there is a need for a standardized approach by which to conduct SbCST in order to effectively evaluate newly built healthcare systems.

This paper describes a standard approach to SbCST, in the post-construction phase of design, when building construction is complete, just prior to opening for patient care. We provide tools for development, implementation, and evaluation of SbCST to probe the built environment and identify LSTs and system inefficiencies prior to patient exposure. This is a collaborative manuscript by authors who have conducted post-construction and renovation SbCST at their respective institutions. Study details and findings of these projects are beyond the scope of this paper [15, 17].

## Conceptual framework

### System errors related to healthcare design

**Fig. 1:**
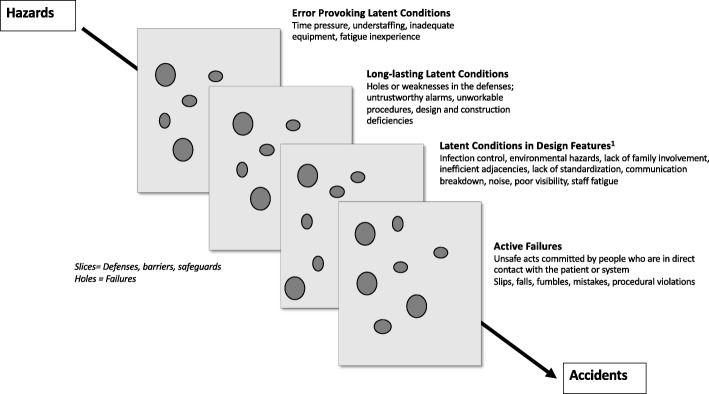
Integration of safe design principles with Reason’s Swiss cheese model of system accidents. Reason’s Swiss cheese model describing how latent conditions and active failures combine to lead to an accident or error [3, 6]. Superscript number (1) indicates evidence-based safe design principles described by AHRQ and CHD [4, 18]

The relationship between system errors and healthcare design can be explained by Reason’s Swiss cheese model which illustrates how defenses, barriers, and safeguards may be penetrated by an accident (Fig. 1) [6]. When multiple “holes” align, there is an opportunity for failure that may impact a patient or staff negatively. Despite exhaustive planning, there are inevitably weaknesses introduced into a system. Decisions made by designers, builders, architects, and management have the potential to introduce either an error-provoking condition, such as inadequate equipment, or a long-lasting weakness, such as design and construction deficiencies [3, 6]. The relationship between safety and healthcare design must be considered as the built environment interacts with people, process, workflow, equipment, and/or technology [3]. Identifying and remediating these latent conditions with SbCST is a proactive means to reduce risk [15]. SbCST provides a clinical context to more effectively examine these interactions by providing teams with an opportunity to actively experience the complexity of patient care delivery not possible with traditional post-occupancy evaluation methods [15].

### Evidence-based safe design principles

Evidence-based safe design principles (EbSDP) put forth by the Agency for Healthcare Research and Quality (AHRQ) and the Center for Health Design (CHD) focus on key evidence-based design considerations to optimize design decisions. These design features are based on available research and have been shown to impact healthcare outcomes [18]. Therefore, we propose that these ten EbSDP described by AHRQ and CHD provide the conceptual framework for SbCST [4, 18]. The ten EbSDP include the following: (1) control and eliminate sources of infection, (2) minimize environmental hazards, (3) optimize adjacencies, (4) support patient/family involvement in care, (5) ensure standardization, (6) reduce communication breakdown, (7) reduce noise, (8) enhance visibility, (9) reduce staff fatigue, and (10) automate where possible. Utilizing the EbSDP as a foundation to design and implement SbCST ensures that testing objectives are evidence-based and offers the ability to detect a wide range of LSTs and system inefficiencies. Each EbSDP is detailed in Additional file 2 [4, 18]. Here, each principle is anchored to a potential latent condition. Key questions that can be utilized to identify LSTs are provided [4, 18].

### Phases of simulation-based clinical systems testing

A standardized approach to conducting SbCST to assess new healthcare post-construction consists of multiple phases (Table 1): the development phase includes identification of a multidisciplinary collaborative workgroup and education on SbCST and needs assessment and process mapping, identification of testing objectives, scenario development, and simulation preparation; the implementation phase includes the simulation event; and the evaluation phase includes scenario debriefing, failure mode and effect analysis (FMEA) scoring, and follow-up of any opportunities for improvement (OFI) implemented following simulations. Documentation tools to support SbCST include a facilitator guide, focused observer questions, a SbCST FMEA scoring rubric, and reporting template (Additional files 1, 3 and 4).

**Table 1:** Suggested timeline for development of simulation-based clinical systems testing (SbCST)

Development phase	
• Stakeholder engagement (6–8 months)	
• Identification of multidisciplinary collaborative workgroup including hospital executive leadership, departmental, and service line leaders	
• Introduce goals and objectives of SbCST	
• Needs assessment, process mapping (3–4 months)	
• Brain storming sessions and process mapping of anticipated concerns related to process, workflow, use of equipment/technology in the new space	
• Scenario development (2–3 months)	
• Simulation team works clinical leaders to design and review simulated scenarios	
• Identification of front-line staff to participate in simulation	
• Simulation preparation (3 months)	
• Collation and organization of testing day materials: rosters, pre-brief presentations, facilitator guides, debrief guides, and FMEA template	
Implementation phase	
• Testing day preparation (1 week)	
• Simulation testing day walkthrough with stakeholders to review scenarios and walk through the testing space	
• SbCST testing day	
• Conduct simulation event	
• Conduct debriefings	
Evaluation phase	
• SbCST testing day	
• FMEA scoring	
• Reporting and follow-up (1 day–1 month post-testing)	
• Create FMEA summary report and follow up with leaders to review what corrective actions have taken place	
• Turnaround time should support leadership implementing changes prior to patient care	

## Development and planning

### Identification of a multidisciplinary collaborative workgroup

SbCST requires engagement of a large multidisciplinary collaborative working group 6–8 months prior to SbCST. Ideally, this collaborative group consists of executive leadership, departmental and service line leaders, institutional operational leaders, ancillary staff, and front-line staff to ensure project support and success (Table 2).

**Table 2:** Description of stakeholder groups

Executive leadership	
• Architect and Design team	
• Chief Executive Officer, Chief Operating Officer, Chief Medical Officer, Chief Nursing and Hospital Operations officer	
Clinical leadership	
• Administrative; Chief Academic Officer, Vice President of Physician Practice, Director of Physician Practice	
• Operational; Manager of Clinical Operations	
• Clinical; Nursing Directors, Assistant Nurse Managers, Clinical Educators, Physician Division Chief, Practice Directors	
Frontline staff	
• Physicians, Nurses, Advanced Practice Providers, Respiratory therapists, Patient Care Technicians, Unit Secretaries	
Ancillary support	
• Information technology, Accreditation, Quality and Safety, Medication Safety, Risk Management, Facilities, Security	

Support from institutional executive leadership is key to ensuring cooperation, involvement, and accountability of departmental, clinical, and service line leaders to promote a culture that values integration of SbCST. These leaders are needed to participate in all phases of SbCST. In the development phase of SbCST, this group’s role is to participate in the needs assessment, process mapping, identification of testing objectives, and scenario design and to identify and ensure staff participation. During SbCST implementation, this group observes each scenario, is present for debriefings, serves as the FMEA scoring team, identifies OFIs, and develops corrective action items in the evaluation phase.

Institutional operational leaders including representation from patient safety and quality, accreditation, infection control, and information/technology should be engaged 3–4 months prior to testing. Members from patient safety and accreditation provide expertise on standards of care and regulatory standards and can support OFIs related to safety threats identified. Infection control can help evaluate how the design and processes being tested impacts infection prevention. Information and technology provide support during SbCST and provide expertise regarding how technology interfaces with staff and patient workflow in the new space. These representatives serve as excellent observers to comment on how things are “intended to be done,” serving as a check and balance to unsafe practices or “shortcuts” adopted by frontline staff in their current workflow. Finally, frontline staff who provide daily patient care and patient/family experience representatives are best suited to identify flaws and concerns as end users.

### Education about SbCST

Messaging of objectives and roles for stakeholders and participants needs to be clear, consistent, and repeated as most stakeholders and participants have never participated in SbCST. Developing a shared mental model that this simulation activity is not to educate, but rather to evaluate the space for LSTs and system inefficiencies is critical for success. Lack of a shared mental model with stakeholders regarding goals and objectives may result in heightened anxiety and stress creating an environment during debriefing that limits honesty and openness of participants.

In-person meetings with clinical leaders should include definition of roles related to the makeup of the collaborative working group, an overview of the role that SbCST plays in the post-construction evaluation, clarification on expectations of clinical leaders, and a synopsis of the SbCST event. A standardized presentation can help educate leaders on the AHRQ and CHD safe design principles reinforcing the conceptual framework for SbCST and align priorities and overarching goals. During these meetings, those who will be responsible for participant identification, and content experts for scenario design can be identified. It is essential to also brief participants on the goals of testing and their role in SbCST. This can be accomplished by face to face meetings, open forums, email, flyers, or videos.

### Needs assessment

A formal needs assessment applying strength weaknesses opportunities and threats (SWOT) analysis [19], KJ Merlin, or KJ Reverse-Merlin exercises should be led by simulationists [20]. These brainstorming sessions and in-person interviews guide clinical leaders and frontline staff to identify high frequency/low acuity events (e.g., routine admissions) and low frequency/high acuity events (e.g., patient decompensation). Staff may identify current unsafe or inefficient workflows, processes, or designs and offer potential new solutions that can be tested and evaluated during SbCST. The needs assessment provides valuable information for the next steps of process mapping, identification of testing objectives, and simulation scenario design.

### Process mapping

Clinical tasks necessary to conduct patient care in the clinical area being tested must be detailed in order to evaluate the specific design principles in question. This can be done through process mapping, a method used in quality improvement initiatives as a way for a team to gain a holistic understanding of a process under review [21]. A process map details each sequence of events related to the process being evaluated [22]. Simulationists work alongside clinical leaders and frontline staff to facilitate the creation of a process map for each clinical situation, process, or workflow that is to be tested. It important that each activity, decision point, necessary personnel, supplies, equipment, and role of participating staff members are clearly identified.

### Identification of testing objectives

Objectives for each simulation event can be developed utilizing the EbSDPs described in Additional file 2. The examples that anchor each EbSDP provide specific themes that can be tested during scenarios. The number of safe design principles evaluated varies depending on the needs of each clinical area. It is important to choose a clinical context that ensures evaluation of a wide range of safe design principles. Activities should prompt performance of tasks needed for clinical staff to engage with design features, process, or workflows that are under investigation [8, 21, 23]. Multiple testing objectives can be met within one scenario. For example, routine movement of a patient through a clinic visit can be used to evaluate safe design principles such as interruptions, excessive walking, adoptability, visibility to/of patients, and location of supplies and equipment.

### Scenario development

Collaboration between the simulation team and stakeholders ensures that the clinical fidelity aligns with SbCST objectives. Simulationists help to anchor each task in the process to a safe design principle providing a platform for multiple elements to be tested within each clinical scenario. Situations that are frequent, urgent, challenging, new to the organization, or high risk should be prioritized [5].

Clinical complexity and the need for complex medical decision making should be minimized to maintain focus on the system and process that support care rather than the medical content of the scenario. The number and length of scenarios depend on how many new areas, processes, or distinct clinical departments are being evaluated with SbCST. The complexity of each individual scenario impacts the duration of the scenario, number of participants, observers needed, and length of the debriefing [5]. Some scenarios may be conducted multiple times to test alternative processes, equipment, or designs [5]. Multiple scenarios may proceed simultaneously within one simulation block to create a virtual unit representing simultaneous episodes of care. These simultaneous scenarios may occur in multiple units and include patient transfer between adjacencies to better represent unit functioning, workflows, and nearby departments.

### Role of participants, observers, and embedded participants

Once scenarios are developed, participants, observers, and embedded participants must be identified. Participants should only fill roles they are normally accustomed to working in and represent varying levels of experience and perspectives [5]. The number of participants may range from 5 to 8 people and will vary based on the clinical context and how many staff/physicians are required to complete the clinical episode care.

Observers should consist of individuals that represent executive leadership, departmental and service line leaders, institutional operational leaders, and front-line clinicians who are experts in their patient care areas. Their role is to observe participants as they engage in workflow and tasks, take notes during simulation, and participate in the debriefing process. We recommend 5–12 observers to witness each episode of care. Observers should be limited in number to minimize overcrowding and disruption of workflow. They should be instructed to not assist with tasks during the scenarios. Providing a tool with questions relating to the safe design principles helps to focus observations on testing objectives (Additional file 3). We propose that observer questions be categorized by broad concepts such as overall design, resource accessibility/workflow efficiency, patient safety, infection control, and patient/family experience. During SbCST, observers should be placed in critical locations within the space being evaluated for monitoring of flaws in specific workflows, designs, or processes.

Embedded participants may be necessary to recreate patient care scenarios and meet objectives. They may act as patients, family members, or representatives of the public in order to create realism and fidelity within the scenario. When appropriate and with advanced preparation, real parents/family representatives may participate. Family members who have served on design committees or members of family advisory councils can provide valuable insight into accessibility of space, efficiency of processes, and wayfinding. Embedded participants and parent/family volunteers should participate in the debriefings to provide their perspective.

### Simulation preparation

In the months prior to SbCST, simulationists must compile event materials including rosters, pre-brief scripts and presentations, facilitator guides, debrief presentations, and focused observer questions.

Scripting and creation of a facilitator guide is essential to standardization of the testing process, staying on task, working within time constraints, and ensuring that all moving parts are in place for each scenario and that testing objectives are met (Additional file 4) [5]. Facilitator guides should include an overview, timeline, participant roles, equipment and supplies needed, a map of space being tested, detailed tasks, and testing objectives. The guide is used by the simulation facilitator to lead participants through the scenario, prompt management decisions, and ensure the performance of tasks related to testing objectives. For example, if evaluating the location of the code cart, scenario facilitator should direct staff to retrieve emergency supplies. “Time warping” may be necessary to maintain focus on testing objectives and stay within time constraints. For example, the scenario facilitator may “time warp” the clinician through their physical exam as medical decision making is not a SbCST objective.

Prior to the SbCST event, the simulation team and leaders involved in scenario development should walk through the space to finalize areas or rooms that will be used during SbCST, identify where observers will be placed, and ensure operational readiness. Lack of appropriate supplies and equipment, which are often delivered to new spaces just prior to opening, can limit the ability to effectively and accurately test systems and processes. Therefore, it is essential that spaces are operationally ready and stocked with the appropriate items necessary to conduct patient care prior to SbCST.

## Implementation

Each SbCST event should begin with participant and observer registration, consent to photography and video recording (if doing so), and a scripted pre-briefing to ensure standardization between all testing events. The pre-briefing should include introductions, review of the event agenda, clarification of each role (participant, observer, embedded participant, and scenario facilitators), overview of the clinical scenarios, and summary of objectives. A reminder that individual performance is not evaluated helps ease anxiety especially when stakeholders and departmental leaders are observing. Psychological safety is promoted by establishing an environment that encourages open and honest discussion. “Think out loud” encourages participants to verbalize thoughts, providing immediate feedback to scenario facilitators and observers which can be explored during debriefing [5].

Once pre-briefing is complete, participants are oriented to the space while observers are placed in predesignated locations. Observers can be distinguished from participants with distinctive clothing, such as a vest or lanyard.

## Evaluation

### Debriefing

There are key differences in debriefing techniques used for SbCST compared to education-based debriefings. Debriefing SbCST requires a facilitator-focused approach where the facilitator elicits reactions for each step in the scenario, guiding the group to identify safety threats and further explore how those LSTs may compromise patient/staff safety, workflow, process efficiency, equipment, and technology. Simulation of virtual units involving simultaneous scenarios is a contrast to simulation for education where one scenario proceeds at a time. As a result of this complexity, multiple facilitators are needed to conduct each simulation block.

Interval debriefings follow each simulation block with the goal of evaluating specific targeted objectives. Time allotted for interval debriefings generally needs to be longer than that allocated for education-based debriefings [17]. Considerations for length include the complexity of simulations, the number of simultaneous scenarios, and the specific objectives to be discussed. Additionally, the prior process simulation experience of the participants will affect the amount of time needed to orient the team. The debriefing should begin with a standardized presentation reminding all parties of the goals of SbCST. It should be reiterated that the debriefing is used to identify LSTs and that while suggestions can be made, identifying corrective actions and solutions for each potential LSTs is not the primary goal, as groups commonly seek to work on possible solutions to perceived issues immediately.

Interval debriefings should focus on the participant experience. If observers and participants debrief as one large group, participants should be asked to state their reactions/comments for any given step in process [17]. Once participants have finished their comments, observers may be invited to ask questions of the participants. Observers should refrain from defensive comments on the justification or reasoning behind decisions made in the design process. Alternatively, some groups may benefit from debriefing participants and observers separately. This may be particularly useful if there are concerns that participants will not feel comfortable to speak openly and honestly in front of the observer group.

Summative debriefings may occur following the entire simulation event where all participants and observers have the opportunity to share observations and experiences related to any simulation block. Time should be allotted for this session where open-ended questioning allows both participants and observers to bring up any additional concerns noted during the simulation event that had not been previously addressed.

There are a variety of approaches to how debriefings may be structured. In a chronological approach, the facilitator utilizes the facilitator guide to elicit reactions for each step in the scenario to identify and explore LSTs. Other strategies to guide the debriefing include use of structured questioning based on each AHRQ/CHD safe design principle. Debriefing techniques such as the PEARLS debriefing framework, advocacy-inquiry method, open-ended questions, and plus delta strategies can be utilized to promote detailed and focused discussions [24, 25]. It is essential to keep the group on track if the conversation becomes tangential or threatens the safety of the debrief environment.

During the debriefing, simulationists or quality and patient safety experts should scribe the discussion into a pre-formatted reporting template to ensure documentation of all issues identified (see Additional file 1). Notes should state each potential safety threat or system inefficiency identified and a description of how that threat may impact patient safety, work flow, or process efficiency.

### FMEA scoring

At the end of each simulation event, a scoring group consisting of departmental, service line leaders, institutional operational leaders, and executive leadership participate in failure mode and effect analysis (FMEA) scoring. FMEA is a proactive risk assessment tool that guides a multidisciplinary team to evaluate a healthcare process [22, 26]. It is a patient safety work product and is therefore confidential, privileged, and not discoverable. At the end of each SbCST event, simulationists use FMEA to guide the team to review, evaluate, and score each potential LST identified during the debriefing. The scoring team reviews each potential LSTS and assigns an occurrence, detection, and severity score which are multiplied to provide a risk priority number (RPN) (Additional file 1) [15, 22, 27]. Each potential LST is then categorized by overall facility design, resources, or processes and workflow issue (Additional file 1) [15].

### Follow-up

A final report with each LST and its score categorized by RPN and LST is then distributed to the scoring team following SbCST (Additional file 1). This scoring group is responsible for identifying OFIs and any corrective actions that need to be taken. The timeline to correction of high-priority items will be dictated by facility opening, resources, and other factors.

## Discussion

SbCST in the preoccupancy post-construction phase of new healthcare design may be utilized to identify potential safety threats related to process, workflow, design, equipment, and technology prior to patient exposure. The purpose of this article is to provide a standardized approach and delineate the stages of planning for SbCST, guiding simulationists from the development phase through implementation and evaluation. The opportunity for application of simulation in healthcare system evaluation is gaining momentum, but lack of a standardized approach makes these projects seem costly, time-consuming, and daunting, particularly to administrators and first-time adopters. The need for individual institutions to reinvent the wheel when preparing to implement a simulation-based design evaluation may limit project success and impact. Without a standard approach and structured conceptual framework to anchor testing objectives, time can be wasted developing tools and recreating templates. Scenarios may be less effective in probing the environment for design features that are known to create latent conditions. Utilizing our stream-lined process will help to maximize time and resource utilization.

Implementing SbCST within the conceptual framework of the AHRQ and CHD helps to focus testing objectives around evidence-based design elements known to impact delivery of care [4]. Utilizing this conceptual framework also allows for a comprehensive evaluation of design where a wide range of LSTs may be detected during a single simulated scenario. This strategic approach further allows for flexibility and adoptability where testing objectives can be tailored to any clinical area.

Creation of new documentation and evaluation tools used during SbCST can be a time consuming and irritative process. A standard set of tools allows for a systematic approach to identify testing objectives, create the clinical context for testing, document findings identified during simulation, organize and record debriefing content, and create comprehensive FMEA reports. These tools included are designed to be applicable in any clinical area such as a patient room, hospital space, outpatient area, or operative space in the post-construction phase of design. This flexibility also allows for modification of testing objectives based on overall project goals and customization for use at any institution choosing to adopt SbCST.

This process encourages active engagement of a large multidisciplinary collaborative working group from senior leaders to front-line staff to better facilitate the exchange of information between leaders, those that deliver care, and architectural teams to more effectively probe the environment. This approach uncovers flaws that may not be identified through other traditional design evaluation methods. While we advocate for early engagement of stakeholders, this is dependent on institutional culture, availability, and competing system priorities. While stakeholders may be engaged in later phases of SbCST planning, early engagement of stakeholders helps to foster buy-in, build testing objectives, engage participants, and maintain accountability for implementing change.

A synthesized way of eliciting information during the debriefing is necessary in order to efficiently conduct the risk assessment and identify LSTs or system inefficiencies. Without structure, debriefings lose focus, veering off into problem-solving, discussing irrelevant process or workflows, or justification of design decisions. Post-construction testing can be an extremely vulnerable process for leaders who have invested considerable time and effort in design and development. Clear communication with stakeholders involves setting expectations prior to testing, and reiterating those goals on the day of testing helps to ensure psychologic safety of the process.

Efficient data collection and gathering of information during debriefing and FMEA scoring is essential to conveying the implications that design elements have on safety and system efficiency. Detailed notes scribed into a preformatted template minimize excessive writing and eliminate transcribing data from multiple sources into a single report. It is essential that the generated FMEA report clearly conveys the context of the threats identified so that these reports can be understood by leaders not present at the SbCST and can be referred to in the future if needed. As these reports are generated, it is important to have clarity on who will take ownership for providing solutions to failure modes with a high-risk priority number. Lack of ownership and accountability limits the integrity of testing if findings are disregarded and opportunities for improvement are deferred. Use of FMEA as a strategy to risk stratify SbCST findings aids leaders in prioritizing and addressing LSTs associated with the greatest risk of harm, mitigating risk prior to patient exposure [15].

A structured and systematic approach to development, implementation, and evaluation of SbCST using the evidence-based safe design principles described by AHRQ and CHD can be adopted by healthcare institutions looking to evaluate a newly designed space. Adoptability and flexibility in documentation and evaluation tools make this type of testing applicable to any type of patient care area.

## Limitations and challenges

Implementing a project of this scale may be difficult to conduct in the face of other operational priorities and competing interests that require time and resources prior to opening a new facility. The need for considerable time, simulation expertise, and resources may limit the feasibility of carrying out this type of testing effectively. Some may question what simulation uncovers that less intensive means of preparation does not address. It is important to stress that administrative planning involves the conceptualization of work, an exercise that is often ineffective in predicting all of the complexities that will actually occur when taking care of patients [10–12]. While a strategic approach to conducting SbCST can streamline project development, considerable buy-in, support from institutional leadership, and a time commitment from both stakeholders and front-line staff are essential to the success of this type of testing.

## Conclusion

This paper describes a systemic approach by which to conduct SbCST and provides documentation and evaluation tools in order to develop, implement, and evaluate a newly built environment to identify LSTs and system inefficiencies prior to patient exposure. Standardization of the approach to development and implementation of SbCST amongst the simulation community has the ability to greatly influence how healthcare facilities are built and tested in the future. Further research is necessary to better determine the impact of SbCST on mitigating patient risk.

## Additional files

Additional file 1: FMEA scoring rubric and reporting template. (PDF 86 kb)

Additional file 2: Safe design principles. (DOCX 20 kb)

Additional file 3: Observer tool. (DOCX 14 kb)

Additional file 4: Facilitator guide. (DOCX 15 kb)

## Data Availability

Not applicable.

## Abbreviations

AHRQ: Agency for Healthcare Quality and Research
CHD: Center for Health Design
EbSDP: Evidence-based safe design principle
FMEA: Failure mode effect analysis
LST: Latent safety threat
OFI: Opportunity for improvement
RPN: Risk priority number
SbCST: Simulation-based clinical systems testing
