# Supplementary material for: Simulation-based clinical systems testing for healthcare spaces: from intake through implementation
Source: Adv Simul (Lond). 2019 Aug 2;4:19. doi: 10.1186/s41077-019-0108-7 (PMC6676572; doi:10.1186/s41077-019-0108-7)
Supplement: Supplementary file 1 — FMEA scoring rubric and reporting template. (PDF 86 kb) [file 41077_2019_108_MOESM1_ESM.pdf]

FMEA SCORING TOOL

Risk Priority Number (RPN) *is calculated by multiplying* ***Severity*** *score by* ***Probability*** *score by the* ***Detection*** *score. Issues are considered significant priorities if* ***RPN*** *is between 32-64 on scale of 1-64.*

	4	3	2	1
**SEVERITY**	**CATASTROPHIC**<br>*Failure could cause death, injury*<br>Patient Outcome:<br>- Death or major permanent loss of function (sensory, motor, physiologic, or intellectual)<br>Visitor Outcome:<br>- A death; or hospitalization of ≥3<br>Staff Outcome:<br>- A death; or hospitalization of ≥3<br>Equipment/Facility damage:<br>- Fire beyond incipient stage; or damages ≥$250,000	**MAJOR**<br>*Failure could cause high degree customer dissatisfaction*<br>Patient Outcome:<br>- Permanent lessening of bodily functioning (sensory, motor, physiologic, or intellectual); or<br>- Increased length of stay or increased level of care for ≥3 patients<br>Visitor Outcome:<br>- Hospitalization of 1-2 visitors<br>Staff Outcome:<br>- Hospitalization of 1-2 staff; or ≥3 staff experiencing lost time, or restricted duty<br>Equipment/Facility damage:<br>- Damages $100,000-$250,000	**MODERATE**<br>*Failure can be overcome, but there is minor performance loss*<br>Patient Outcome:<br>- Increased length of stay or increased level of care for 1-2 patients<br>Visitor Outcome:<br>- Evaluation, treatment of 1-2 visitors<br>Staff Outcome:<br>- Medical expenses, lost time, or restricted duty for 1-2 staff<br>Equipment/Facility damage:<br>- Damages $10,000-$100,000; or<br>- Fire, at/smaller than incipient stage	**MINOR**<br>*Failure not noticeable to customer, no effect on delivery of service*<br>Patient Outcome:<br>- No injury, nor increased length of stay, nor increased level of care<br>Visitor Outcome:<br>- Evaluated, but no treatment<br>Staff Outcome:<br>- First aid only, no lost time, or restricted duty<br>Equipment/Facility damage:<br>- Damages <$10,000; or loss of utility without adverse patient outcome
**PROBABILITY OR OCCURRENCE**	**FREQUENT**<br>*Likely to occur immediately or within a short period (may happen several times in 1 year)*	**OCCASIONAL**<br>*Probably will occur (may happen several times in 1 to 2 years)*	**UNCOMMON**<br>*Possible to occur (may happen sometime in 2 to 5 years)*	**REMOTE**<br>*Unlikely to occur (may happen sometime in 5 to 30 years)*
**DETECTION**	**ALMOST IMPOSSIBLE**<br>*No known controls are available to detect failure mode*	**REMOTE**<br>*Remote likelihood that current controls will detect failure mode*	**MODERATE**<br>*Moderate likelihood that current controls will detect failure mode*	**HIGH**<br>*High likelihood that current controls will detect failure mode. Reliable detection controls are known with similar processes*

Threat Detected	LST Category	Potential Failure Effect	Action Recommended by Scoring Team	Severity	Occurrence	Detection	RPN	30-day Post Simulation Action Taken
What is the process step, change or feature under investigation?	**[Resource Issue, Process/Systems Issue, Facility Issue, Clinical Performance issue]**	*What is the impact on the customer if this failure is not prevented or corrected?*	*What are the recommended actions for reducing the occurrence of the cause or improving detection?*					*What action will be taken in relation to this finding and what will the follow-up plan be?*

Threat Detected	LST Category	Potential Failure Effect	Action Recommended by Scoring Team	Severity	Occurrence	Detection	RPN	30-day Post Simulation Action Taken
			-					

Additional File 1: Appendix D. FMEA Scoring Rubric and Reporting Template
