# Supplementary material for: Simulation-based clinical systems testing for healthcare spaces: from intake through implementation
Source: Adv Simul (Lond). 2019 Aug 2;4:19. doi: 10.1186/s41077-019-0108-7 (PMC6676572; doi:10.1186/s41077-019-0108-7)
Supplement: Supplementary file 2 — Safe design principles. (DOCX 20 kb) [file 41077_2019_108_MOESM2_ESM.docx]

| **AHRQ and CHD Evidence-based Safe Design Principles** ^1^ | |
| --- | --- |
| **Design Framework**  **Latent Conditions** | **Examples** |
| **Standardization**  The presence of multiple locations of equipment and supplies as well as multiple ways of doing things adds to the cognitive burden on staff and increase chances of error. Requiring reorientation with each activity, wastes time and injects opportunity for distraction and error in decision making. | - Did you notice any difficulty getting all necessary equipment and supplies to the patient(s) due to insufficient space or poor room layout? - Was the location of equipment and supplies accessible during high-risk care episodes? - Is there sufficient space and effective layout to adapt to different patient care needs? - Did providers know where to access the emergency equipment? - Did the location of equipment and supplies create delays in patient care? |
| **Staff Fatigue**  Fatigue has a negative impact on alertness, mood, and psychomotor and cognitive performance, all of which are linked to active failures.  Unit layout should minimize extensive walking to hunt and gather supplies, people, and should limit frequent work interruptions. | - Does the layout require extensive walking to gather supplies or people? - Did the layout result in frequent work interruptions? - Did you notice any concerns related to provider fatigue during patient care? - Does location of storage areas allow for efficient workflow? |
| **Enhance Visibility to Patients**  Staff depends on visual and auditory cues in order to respond to the needs of patients and prevent adverse events such as falls.  Building design should facilitate visual access to patients. | - Did the overall design impact visibility? |
| **Reduce Noise**  High noise levels result in staff stress, exhaustion, and burnout and also impact patient sleep and healing. | - Was there privacy in clinical staff work stations? |
| **Reduce Communication Breakdown**  Communication discontinuities and breakdowns and lack of timely access to critical information may adversely affect patient safety. | - Does the physical environment support effective teamwork and communication? |
| **Control/Eliminate sources of infection**  Most healthcare-associated infections are contact- transmitted to patients from the hands of healthcare staff and contact with contaminated surfaces. | - Is there an adequate physical separation and/or isolation method (e.g., separate soiled workroom, supply chain flow separation) in the clinic layout to prevent contamination of clean supplies and equipment? |
| **Minimize environmental hazards**  Hazards in healthcare environments can result in slips, trips, and falls among patients as well as staff.  Design should limit the placement of equipment, IV poles, furniture in the path of movement. | - Was there unnecessary crowding of equipment and/or personnel during patient care? |
| **Automate where possible**  Automation of certain tasks increases accuracy and reduces probability of error.  Design should minimize handoffs of patient or transfer of information. | - Did you notice any risk to patient safety due to difficulty obtaining vital patient information? - Did you notice any threats to incorrect patient identification? |
| **Support patient and family involvement in care**  Involvement and participation of patients and family members can help to reduce adverse events such as errors and falls. | - From a patient and family experience perspective, does the wayfinding seem intuitive/easy-to-follow? - Did you notice any threats to maintaining patient privacy? - Easily accessible communication system (e.g. telephone, intercom) for staff between patient room and other healthcare spaces (e.g. nursing station) |
| **Consider Adjacencies^2^**  Consider vertical and horizontal adjacencies to optimize processes, patient movement, and distribution of materials, equipment, and supplies.  Design should limit cross traffic of patients and materials, food, waste, supplies. | - Was the transfer of patients safe? - Did you notice any risks associated with transporting patients through the building? (e.g., ample corridor width, minimal turns, wide doorways, open layout, elevators with ample spaces to accommodate stretchers, etc.) - Do you have any concerns for the ambulation of obese or mobility impaired patients? |

Appendix A. AHRQ and CHD evidence-based safe design principles

^1^ Adopted from AHRQ and CHD evidence based safe design principles [4, 18].

^2^ Adjacencies refer to areas that directly support patient care and may include (but is not limited to) diagnostic areas such as radiology, laboratory, or clinical support areas such as clean supply, equipment rooms, soiled utility rooms, nourishment rooms and/or care team workstations.

AHRQ-Agency for Healthcare Research and Quality, CHD-Center for Health Design
