# Supplementary material for: Simulation-based clinical systems testing for healthcare spaces: from intake through implementation
Source: Adv Simul (Lond). 2019 Aug 2;4:19. doi: 10.1186/s41077-019-0108-7 (PMC6676572; doi:10.1186/s41077-019-0108-7)
Supplement: Supplementary file 3 — Observer tool. (DOCX 14 kb) [file 41077_2019_108_MOESM3_ESM.docx]

| **Safe Design Goals** | **QUESTIONS** | **COMMENTS** |
| --- | --- | --- |
| **Overall Design** | Are there inefficiencies in unit layout?  Are there visual distractions that impact workflow?  Does the unit layout minimize extensive walking? |  |
| **Resource Accessibility& Workflow Efficiency** | Does the location of equipment and supply storage impact the timeliness of patient care?  Does the location of storage areas impact workflow?  Does the flow and accessibility of supplies support high-acuity care episodes? |  |
| **Patient Safety** | Does the physical environment impact teamwork and communication?  Does the layout support the ability to adapt to patient care needs such as resuscitation, and or tests/procedures?  Does the overall design impact visibility?  Did you notice any risks associated with transporting patients through the building? |  |
| **Infection Control** | Does the overall design impact infection risk or cross-contamination?  Does the design limit cross-traffic of patients with supplies and equipment?  Is there an adequate physical separation or isolation methods (e.g., separate soiled workroom, supply chain flow separation) to prevent contamination of clean supplies and equipment? |  |
| **Patient and Family Experience** | Does the overall layout support the privacy of patients?  Does the design support the intended movement of families and patients through the space?  Does the design of clinician-centered areas safeguard PHI and support HIPPA? |  |

Appendix B: Focused observer questions
