# Supplementary material for: Simulation-based clinical systems testing for healthcare spaces: from intake through implementation
Source: Adv Simul (Lond). 2019 Aug 2;4:19. doi: 10.1186/s41077-019-0108-7 (PMC6676572; doi:10.1186/s41077-019-0108-7)
Supplement: Supplementary file 4 — Facilitator guide. (DOCX 15 kb) [file 41077_2019_108_MOESM4_ESM.docx]

| **SbCST Facilitator Guide** | | | |
| --- | --- | --- | --- |
| **OVERVIEW**  **Date**:  **Phase/Specialty:**  **Scenario:** | **SIMULATION CENTER STAFF**  **Facilitator:**  **Simulation Technician:** | | |
| \| **SCHEDULE** \| **Approximate Start Time** \| **Timeframe** \| \| --- \| --- \| --- \| \| **Scenario Pre-Brief** \|  \|  \| \| **Scenario Time** \|  \|  \| \| **Move to Classroom** \|  \|  \| \| **Debrief** \|  \|  \| \|  \| \| \| | **PARTICIPANT ROLES** | **EMBEDDED PARTICIPANTS** | |
|  |  | **OBSERVERS** | |
| **SCENARIO OUTLINE** | **PATIENT BACKGROUND** | | |
|  | **EQUIPMENT/SUPPLIES** | | **OPTIONAL SYSTEM REPS** |
| **VITAL SIGNS & PATIENT STATUS**   \|  \|  \| \| --- \| --- \| |  |  |  |
| **Floor Map of Clinical Area** | | | |
| **SIMULATION PROCESS TESTING GOALS & NOTES**   \| **Overall Design** \| **Resource accessibility/workflow efficiency** \| \| --- \| --- \| \| **Patient Safety** \| **Patient and Family Experience** \| \| **Infection Control** \|  \|   **SCENARIO**   \| **STEP-BY-STEP PROCESS** \| **PROCESS BEING TESTED** \| **OBSERVATION NOTES** \| \| --- \| --- \| --- \| \|  \|  \|  \| \|  \|  \|  \| \|  \|  \|  \| \|  \|  \|  \| \|  \|  \|  \| \|  \|  \|  \| | | | |

Appendix C: Facilitator Guide
